# Innovative Approach for Enhancing Testing of HIV, Hepatitis B, and Hepatitis C in the General Population: Protocol for an Acceptability and Feasibility Study (BaroTest 2016)

**DOI:** 10.2196/resprot.9797

**Published:** 2018-10-12

**Authors:** Nathalie Lydié, Leila Saboni, Arnaud Gautier, Cécile Brouard, Stéphane Chevaliez, Francis Barin, Christine Larsen, Florence Lot, Delphine Rahib

**Affiliations:** 1 Sexual Health Unit Santé publique France Saint-Maurice France; 2 HIV, Hepatitis B/C and STI Unit Santé publique France Saint-Maurice France; 3 Surveys Unit Santé publique France Saint-Maurice France; 4 Centre National de Référence des Hépatites B, C et Delta, Laboratoire de Virologie Hôpital Henri Mondor Université Paris-Est Créteil Créteil France; 5 Centre National de Référence du VIH & Inserm U1259 CHU Bretonneau Université François-Rabelais Tours France

**Keywords:** home self-sampling, dried blood spot testing, feasibility studies, HIV infections, hepatitis B, hepatitis C, cross-sectional studies

## Abstract

**Background:**

Despite substantial screening for HIV, hepatitis B virus (HBV), and hepatitis C virus (HCV) infections in France, a great number of infected persons remain undiagnosed. In this context, Santé publique France experimented with a new screening approach for HBV, HCV, and HIV infection, based on home self-sampling using dried blood spot (DBS) for blood collection.

**Objective:**

The objectives of the BaroTest study were to assess the acceptability and feasibility of this approach and to update the prevalence estimates of HBV, HCV, and HIV infections in the general population.

**Methods:**

Participants were enrolled using the 2016 Health Barometer, a national cross-sectional telephone survey based on a large representative sample of the general population aged 15 to 75 years (N=15,000). Upon completion of the questionnaire, any participant in the Health Barometer aged 18 to 75 years, having medical health insurance, and not under guardianship was invited to receive a self-sampling kit delivered by standard postal mail and to return the DBS card to the laboratory. The laboratory was then responsible for reporting the results to the participants. Acceptability of the protocol was based on the percentage of eligible individuals agreeing to receive the self-sampling kit, on the proportion of people returning the DBS card, and on the proportion of participants out of the total eligible population. The feasibility of the approach was based on the number of participants with adequately filled blood spots and the number of participants with blood spots for which at least one virological analysis could be performed. A complex system of reminders was implemented to increase the participation rate. Accordingly, we assumed that 35.00% (4900/14,000) of eligible persons would accept and return their DBS card. As the highest expected prevalence was for HBV infection, estimated at 0.65% in 2004, 5000 persons would make it possible to estimate this prevalence with an accuracy of approximately 0.22%. All indicators can be analyzed according to the characteristics of the participants collected in the Health Barometer questionnaire. BaroTest was approved by the French Ethics Committee (November 11, 2015) and the Commission on Information Technology and Liberties (December 24, 2015). The study has been registered by the French medical authority under number 2015-A01252-47 on November 10, 2015.

**Results:**

The results on acceptability and feasibility are expected in the last quarter of 2018 and those on the prevalence estimates in the first semester of 2019.

**Conclusions:**

The BaroTest results will help to inform new strategies for HIV, HBV, and HCV screening, and the Health Barometer provides a reliable updated assessment of the burden of HBV, HCV, and HIV infections in the general population in France while reducing the costs typically associated with this type of research.

**Registered Report Identifier:**

RR1-10.2196/9797

## Introduction

France is a low-endemic country for HIV infection, chronic hepatitis B virus (HBV), and chronic hepatitis C virus (HCV) infections, with prevalence in the general population estimated at 0.29% (in 2013), 0.65% (in 2004), and 0.42% (in 2011), respectively [1-3]. Prevalence of these infections is greater in subgroups at risk. For example, HIV prevalence was 17% in a community sample of men who have sex with men (MSM) in Paris [4], 13% among intravenous drug users [5], and 1.6% among Afro-Caribbeans living in the greater Paris area [6]. Anti-hepatitis B core antibody prevalence increased with the hepatitis B virus surface antigen (HBsAg) endemic level of the country of birth and was higher in the individuals with a history of intravenous drug use (50.1%) and among MSM (29.4%) [2]. The anti-HCV prevalence varied from 63.8% among people who reported ever injecting a drug and 1.8% among immigrants [3].

Screening activity is substantial; the annual rate of tests performed in public and private medical laboratories being 58 per 1000 inhabitants for HBV, 55 per 1000 inhabitants for HCV, and 80 per 1000 inhabitants for HIV [7,8]. Screening has expanded in recent years, with the introduction of rapid diagnostic tests performed by nonmedical staff (since 2012 for HIV, since 2016 for HCV, and in 2018 for HBV). However, a great number of infected persons remain undiagnosed, estimated at 155,000 people in 2004 for HBV, 74,000 in 2014 for HCV, and 24,800 in 2013 for HIV [9-11]. Accordingly, they do not benefit from the currently available effective antiviral treatments. Indeed, since 2014, direct-acting antivirals (DAAs) represent a major turning point in the treatment of hepatitis C, with more than 90% of treated patients being cured [12]. Access to DAAs is now free for all HCV-infected patients in France, irrespective of liver fibrosis stage, and this raises the hope that the epidemic will be controlled in the medium term in the country. Antiviral treatments for HIV and HBV keep viral replication under control in the majority of infected patients. Achieving sustained virological response with DAAs for hepatitis C and maintaining an undetectable viral load with HBV or HIV treatment are essential steps to reducing the risk of morbidity and mortality and to preventing the risk of transmission [13-15].

French screening strategies have been modified in recent years to foster earlier screening for HIV, HBV, and HCV infections and reach populations unaware of their infection. Recommendations strengthened the frequency of HIV screening in the key populations (eg, HIV testing every 3 months for MSM, once a year for drug users and migrants from sub-Saharan Africa). In addition, a complementary approach to screening in key populations was implemented to enable people who are unaware of their infection to be diagnosed and to reduce the hidden epidemic. The HIV, HBV, and HCV testing *at least once in the lifetime* is now proposed to individuals aged 15 years irrespective of their exposure risk (universal screening) [16]. Since 2014, combined screening for HIV, HBV, and HCV infections has also been recommended [17]. In this context, Santé publique France, the national public health agency, experimented with a new combined screening approach for HBV, HCV, and HIV infections, based on home self-sampling using the dried blood spot (DBS) for blood collection with the *BaroTest* study.

## Methods

### Objective

The primary objective of the *BaroTest* study was to assess the acceptability and feasibility of screening for HBV, HCV, and HIV infections using home-based self-collected blood samples on filter paper.

The secondary objective was to update the prevalence estimates of HBV, HCV, and HIV infections as well as undiagnosed infections in the general population.

### Sampling and Study Enrollment

**Figure 1:**
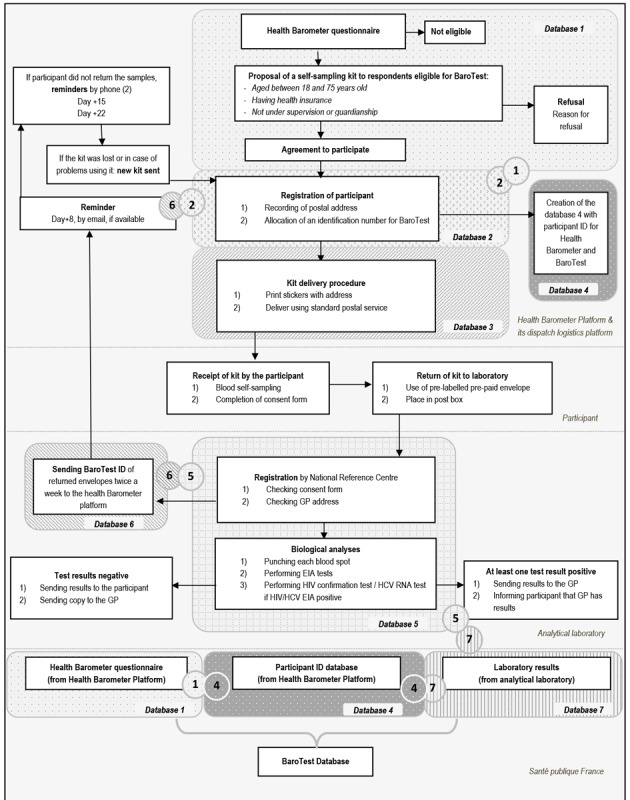
Operational flowchart, BaroTest Study, 2016. EIA: enzyme immunoassay; GP: general practitioner; HCV: hepatitis C virus.

**Figure 2:**
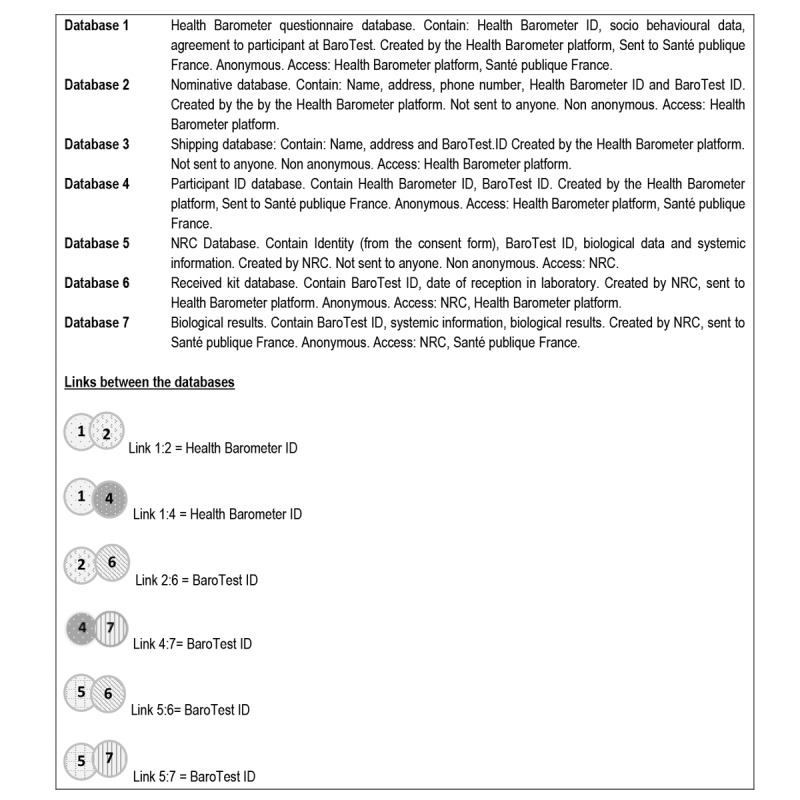
Legend for flowchart in Figure 1.

Participants were enrolled using the 2016 Health Barometer, a national cross-sectional telephone survey based on a representative random sample of the general population (15,000 individuals) aged 15 to 75 years living in mainland France (Figures 1 and 2).

The sampling method for the 2016 edition was identical to that developed for the 2014 Health Barometer [18]. Fixed-line and mobile phone numbers were randomly generated, with 1 individual being randomly selected from eligible members of the household. If the selected person agreed to answer the questionnaire, a unique 9-digit Health Barometer identifier was attributed to them. In case of refusal, the selected individuals and their household were not replaced.

The 40-minute-long phone survey questionnaire was administered by a trained investigator. It included questions evaluating general health, hygiene and protective habits, sexual and preventive behaviors, contraception, knowledge of vector-borne diseases and their prevention, opinions and attitudes toward vaccination, and attitudes toward HBV, HCV, and HIV screening.

### Study Population

Any participant in the 2016 Health Barometer aged 18 to 75 years, having medical health insurance, and not under guardianship was eligible for the BaroTest study.

### Study Design

Upon completion of the Barometer phone–based questionnaire, all those eligible for BaroTest were invited to receive a self-sampling kit for HBV, HCV, and HIV testing delivered by standard postal mail.

The collective and individual benefits of screening, as well as the overall objectives of the survey were explained. When a participant declined this invitation, the reason was recorded. If accepted, the investigator:

- registered the first name and surname of the participant, along with the address to which the self-sampling kit would be sent;
- informed the participant that personal information would be recorded separately from information collected during the phone questionnaire and would only be retained for the period covering the delivery of the self-sampling kit, home self-sampling, and the return of the sample to the laboratory for testing;
- invited the participant to provide an email address and a telephone number so that a reminder could be sent in case the laboratory did not receive the dried blood sample card within 15 days of the dispatch date of the kit; and
- provided the participant with a telephone number and an email to be used to contact the investigator, in case of nonreceipt of the kit, difficulties with its use, or for any other issues concerning the study.

**Components of the kit.**

- An information letter outlining the objectives and details on voluntary participation in the BaroTest. This letter also listed the telephone numbers of various helplines (AIDS Info Service and Hepatitis Info Service) for any questions related to HIV or AIDS, hepatitis B virus (HBV), and hepatitis C virus (HCV)
- A 2-page consent form to be signed and completed with the participant’s name, date of birth, postal address to receive the results of the tests, and the contact details of a general practitioner (GP) of his or her choice to whom the results would also be sent
- The self-sampling kit, with detailed instructions for blood-sample collection; 2 single-use safety lancets; a prenumbered filter paper card (Whatman 903 FTA cards) with 5 preprinted circles zones, named spots (diameter: 6 mm); a disinfection cotton pad; and a small adhesive sensitive skin bandage
- A sealable plastic bag to protect the dried blood spot (DBS) including a desiccant packet to remove any moisture from the DBS card
- A bubble pouch for the 2 lancets to be returned with the DBS to ensure elimination of clinical infectious waste
- A prestamped and preprinted rip-resistant envelope addressed to the National Reference Centre (NRC), the laboratory in charge of HIV, HCV, and HBV analyses

The BaroTest participant was randomly assigned a prenumbered self-sampling kit, thereby defining his or her identifier number in the study (BaroTest ID). The kit was sent inside a resealable cardboard box in a large and plain envelope within 3 days of the completion of the Barometer questionnaire (Textbox 1).

After receiving the kit, the participant performed a capillary whole-blood sampling procedure by drawing blood from the fingertip with the lancet and depositing 1 large drop of free-flowing blood onto each of the 5 preprinted circles on the card, named *spots*. A spot is the area within the 6-mm diameter circle that is supposed to be filled with blood.

Once the self-sampling was performed and the blood sample card dried at room temperature (at least 3 hours), the participant inserted the DBS card into the sealable plastic bag with the desiccant packet, put the closed plastic bag into the resealable cardboard box along with the completed signed informed consent form and the bubble pouch with the 2 lancets, put the cardboard box in the return rip-resistant envelope, and sent it by mail to the National Reference Centre (NRC). Packaging and shipping followed international shipping guidelines and regulations for the Transport of Infectious Substances (WHO/HSE/GCR/2012.12).

The phone numbers of the HIV or AIDS and viral hepatitis national hotlines were mentioned in the consent form of the participants. These hotlines are open every day throughout the year. The staff was trained to address any sexual health question, including the topic of violence. The respondents were trained before the survey to answer any basic questions regarding the study. In addition, a study-specific email address was opened to the participants during the study to contact the study coordinator for any difficult question and to manage any adverse event that was transmitted via the hotlines.

### Reception and Analyses of the Dried Blood Spot

Upon receipt of the envelope at the NRC, the following data were recorded in the NRC database: the BaroTest ID, the date of receipt, the name and address of the participant, the name and address of the general practitioner (GP), the status of the consent form (enclosed or not, signed or not, completed or not), and status of the DBS card (enclosed or not filled or unfilled). The DBS cards were assessed by a trained laboratory technician for both validity and amount of blood. The number of spots categorized as *empty*, *correctly filled*, or *incorrectly filled* was recorded in the NRC database.

Hepatitis B and C serological tests were based on the detection of the HBsAg and total anti-HCV antibodies, respectively. For this purpose, a single spot was eluted in 1 mL phosphate buffered saline with gentle agitation for 1 hour at 4°C and then centrifuged at 36,220 *g* for 1 min before use.

Qualitative HBsAg detection was performed by means of an automated enzyme immunoassay (EIA; VIDAS HBsAg Ultra, BioMerieux, France).

Detection of total anti-HCV was conducted by means of a third-generation EIA (aHCV Vitros ECi, Ortho-Clinical Diagnostics, Raritan, New Jersey, USA). If anti-HCV was positive, HCV RNA was detected with the Abbott RealTime HCV assay (Abbott Molecular, Des Plaines, Illinois), a real-time polymerase chain reaction–based method. Briefly, a second 6-mm spot was eluted into 1.5 mL Lysis buffer from Abbott at 56°C with gentle agitation for 30 min and then centrifuged at 36,220 g for 1 min before use.

For HIV analyses, a punch, 6 mm in diameter, from the DBS was placed in 150 µL of 0.01 M sodium phosphate buffer containing 10% bovine serum albumin and 0.05% Tween 20 (PBS-BSA-TW) and then incubated overnight at 4°C. The BioRad fourth-generation enzyme-linked immunosorbent assay (ELISA; Genscreen Ultra HIV Ag-Ab combo assay) was used to detect both anti-HIV and p24 antigen. The eluted samples were directly transferred to ELISA microplates (75 µL per well). Subsequent steps were performed according to the manufacturer’s recommendations.

If the HIV test proved positive, a confirmatory test (Western Blot, HIV Blot 2.2, or MP Diagnostics) was performed on the second spot. As described above, a punch 6 mm in diameter was placed in 1.4 mL of PBS-BSA-TW and then incubated overnight at 4°C. The eluted volume was brought up to a total of 2 mL with the addition of a dilution buffer from the Western Blot kit and incubated with the strip. Subsequent steps were performed according to the manufacturer’s recommendations.

All the results were validated by a medical biologist at the NRC and then recorded in the laboratory database.

### Reporting to Participants

The reporting procedure was explained in the participant consent form and differed according to the results (positive or negative). If all 3 tests for HIV, HBV, and HCV proved negative, the NRC informed the participant and the GP by postal mail. However, in this correspondence, it was also indicated that tests conducted using DBS do not have the same performance levels as conventional screening methods based on plasma or serum collected on venous puncture performed by specialists and, consequently, are not as accurate. Participants were advised that in case of recent exposure at risk of infection, they should contact their GP for additional biological testing, if required.

When at least one of the tests was positive or showed a limit result, the NRC sent the results of all tests to the GP of that participant only (ie, not to the participant) under confidential cover, along with a letter informing the GP that the participant had been invited to obtain the results and advising the GP to verify the positive result using a conventional standard screening method. The NRC contacted the participant by postal mail inviting him or her to contact the GP designated in the consent form to obtain the results of all tests.

### Reminders to Participants

Eight days after the self-sampling kit was posted to the participants, an email was automatically sent to those who had provided an email address, asking them to confirm reception or not. Those who did not receive the kit were invited to contact the survey institute for help and information.

The NRC sent a list of BaroTest identifier codes (reflecting individual participants) corresponding to the returned samples, twice a week to the survey institute by email. For each BaroTest ID listed, details were provided on what exactly had been received. In case of incomplete submissions (eg, no filter paper or no consent form) or missing information (eg, unsigned consent form or incomplete contact details), the survey institute telephoned the participant to enquire whether he or she required another sampling kit or consent form to be sent.

Participants were contacted by the survey institute if the DBS sample was not received by the NRC within 15 days after the self-sampling kit was sent. If the participant did not respond after 10 attempts, a voice message and email reminder (for those who had provided an email address) were sent. A second telephone reminder took place 10 days after the first one if no DBS reached the laboratory by that date. Similarly, a voice message was left after 10 attempts, and an email was sent if the person’s email address was available.

### Statistical Analyses

#### Number of Subjects Included and Number of Dried Blood Spots Expected

It was planned to include 15,000 people aged 15 to 75 years in the 2016 Health Barometer, approximately 14,000 of who were aged between 18 and 75 years.

The proportions of eligible individuals agreeing to participate in BaroTest and those who sent back the self-sampling kit were estimated on the basis of previous studies. In 2006, 76% of respondents in the French Sexual Behavior Survey agreed to receive a kit for the detection of Chlamydia trachomatis [19], and in a meta-analysis, Jamil et al calculated an average acceptance rate of 79% [20]. In the experiment described by Fisher et al [21], 62.5% of HIV-negative homosexuals seeking care in a medical setting accepted the proposed home self-sampling kits for sexually transmitted infection or HIV. Of these, 77.5% used them and returned their samples. In our BaroTest study, we assumed that half of the 14,000 eligible participants in the Health Barometer will agree to receive the kit for the BaroTest study. This percentage was lower than that observed in the studies mentioned above, as we considered the nature of the infection screened and the self-puncture used. Among the 7000 individuals who will receive the kit, we assumed that 70.00% (4900/7000) will return their DBS on filter paper, corresponding to 35% of the eligible Health Barometer participants. The 70.00% (4900/7000) return rate was based on the 68% and 62% participation rates in 2 French home-screening studies for chlamydia infections [19,22] and on the hope that our thorough system of reminders would maximize the rate of return.

As the highest expected prevalence was for the HBsAg, estimated at 0.65% in 2004, 5000 persons would make it possible to estimate this prevalence with an accuracy of approximately 0.22%.

### Definitions and Assessment of Acceptability and Feasibility

Acceptability of this screening protocol was based on the percentage of eligible individuals agreeing to receive the self-sampling kit (acceptance rate), on the proportion of people returning the DBS (return rate), and on the proportion of participants out of the total eligible population.

The feasibility of self-sampling testing was based on the number of participants with adequately filled blood spots and the number of participants with blood spots for which at least one virological analysis could be performed.

The amount of blood received was assessed by the number and size of the blood spots. Spots were classified into 3 categories: (1) correctly filled, (2) incorrectly filled, and (3) empty blood spots. In a *correctly filled blood spot*, the spot was completely filled with approximately 10 μL of whole blood. An *incorrectly filled blood spot* was defined as either a blood spot with less than 10 µL of whole blood or an overfilled spot. An *empty blood spot* was defined as a completely empty blood spot.

Prevalence was defined as the proportion of persons testing positive among the population tested. These data were extrapolated to the general population considering the BaroTest participation rate and characteristics of the participants.

The proportion of infected persons unaware of their infection was defined as the proportion of people who reported either that they had never been previously screened or that their last test was negative in the Barometer, among those testing positive.

All indicators were analyzed according to the characteristics of the participants collected in the Health Barometer questionnaire:

- Sociodemographic characteristics: sex, age, country of birth, level of education, and employment status
- Past at-risk exposure to HIV, HBV, and HCV infection: transfusion; drug use; tattooing or piercing performed without single-use equipment; surgical, dental, or nursing care; or prolonged stays in high endemic areas (eg, Africa, Asia, or the Middle East)
- Awareness of hepatitis B and C and HIV serologic statuses: screening history and date and results of the last screening

The characteristics of those who refused to participate in the BaroTest and those who agreed to participate but did not return a biological sample were also analyzed.

### Ethical Statements and Data Confidentiality

#### Information Provided to the Participant

The objectives and methods of the BaroTest study as well as the rights of participating individuals were presented both at the end of the telephone interview and in the letter of information sent with the self-sampling kit. At both times, the potential participant was informed that his or her participation was voluntary and that he or she was fully entitled to refuse participation with no prejudice of any kind. Moreover, mentioned in the letter of information was the right to object, to access, and to rectify participant information held electronically, in accordance with the provisions of Law Number 78-17, January 06, 1978 (French Data Protection Act).

#### Participant Consent

Consent to participate in the BaroTest was obtained at 2 points:

- Initial oral consent was obtained by telephone at the end of the Health Barometer interview after information was provided on the objectives of the study and guarantees given about of anonymity and confidentiality of the records.
- Written consent was obtained via the consent form sent with the self-sampling kit. On this form, it was explained that completed informed consent was required to be able to participate in the BaroTest and to be informed of the results. The right of opposition to, access to, and rectification of all data collected relating to the BaroTest was explained. Participants were advised to contact the survey institute in charge of managing the personal data files if they wished to exercise this right. Participants were also informed that the NRC would store completed consent forms for 15 years and then destroy them.

#### Reporting Screening Process

In accordance with the French law, the reporting process was dependent on the results of the tests (positive or negative, see above). To optimize reporting, several reminder procedures were implemented to minimize the risk that participants would return envelopes with missing or incomplete contact addresses. When returned consent forms only included clear contact details for the GP, screening results were sent to him or her with an explanatory letter. The GP was then responsible for sharing the results with the participant.

#### Confidentiality and Data Flow

For those who agreed to participate in the BaroTest, the survey institute was in charge of the recording of personal data at the end of the interviews as well as of sending the self-sampling kits and managing reminders.

To ensure the safety and confidentiality of personal data, a data file segregation procedure was implemented, whereby the matching of Health Barometer data (answers given during the phone questionnaire) with BaroTest data (testing results) was made impossible. Therefore, at no time did the survey institute receive or keep any of the results from the participants’ tests. The NRC was in charge of managing personal contact data for the participants to ensure delivery of their test results. No transfer of personal data took place between the NRC and other partners (the survey institute, Santé publique France). NRC only sent the ID list of the BaroTest kits it received to the survey institute to manage reminders.

At the end of the study, Santé publique France received the following:

- From the survey institute: (1) an anonymized file of the answers to the Health Barometer 2016 questionnaire with Health Barometer identifiers and (2) a match list of BaroTest and Health Barometer IDs.
- From the NRC: (1) a file containing the BaroTest IDs, (2) the corresponding testing results, and (3) the following information: quality of DBS, date of self-sampling, date of receipt of the self-sampling envelope, GP contact information, and date the results sent by the NRC. This file did not include any personal data. Santé publique France was therefore in possession of 3 anonymized files that it merged using the BaroTest and Health Barometer identifiers to obtain a single database. Following the generation of this database, the BaroTest IDs were definitively deleted.

### Timeline

Inclusion in BaroTest took place between January and July 2016. Following the reminder campaign and to account for late receipt of samples, it was decided that the NRC could analyze and report the results to the participants until December 31, 2016.

## Results

The results on acceptability and feasibility of screening using home-based self-sampling are expected in the last quarter of 2018 and those on the prevalence estimates of HBV, HCV, and HIV infections in the first semester of 2019.

## Discussion

The objectives of BaroTest were to assess the acceptability and feasibility of joint screening for HIV, HBV, and HCV infections using home self-sampling of capillary blood on filter paper and to both assess the number of persons affected in the general population and describe their characteristics.

The BaroTest was linked to a randomized telephone survey called the *Health Barometer*, which uses a complex call protocol to increase the likelihood of interviewing hard-to-reach individuals and to achieve a high response rate. According to the current French bioethics law, the individuals without health insurance were unable to participate in the study, but their number was probably low. Indeed, in France, universal health coverage is accessible to low-income individuals or without any resources as well as the state medical aid for foreigners with an irregular administrative situation. In addition, it is uncertain that all participants with positive screening tests will consult the physician mentioned in their consent form to get their results. The main objective of this study was to target screening to enlarge the awareness of the serological status among the general population. We agree that it would have been efficient to link those tested positive to care. In fact, we proposed to the ethics committee that the medical staff of the NRC phone the participant when one of the tests was found positive. The participant could have been informed, counseled, and referred for control to a physician or the nearest screening center. However, the ethics committee refused this proposal because, in France, positive results have to be given by the GP to the patient face-to-face. That is why we sent the results (when one of the tests was positive) to the GP mentioned in the informed consent and informed the participant by mail that the results of the tests were available with the GP. We checked the address of the GP mentioned in the consent, but there was no mechanism to remind participants to contact their GP for results. However, results of studies on home sampling [23] and self-testing [24] showed that participants seem to base their follow-up behavior on the result of the test, and after an abnormal result, most of them seek medical care. We also assumed that most of the patients will be contacted by their physicians after receiving the positive results if they do not do so themselves. The whole reminder system was based on information feedback between the NRC and the logistic platform. Reminders were sent only to people who had not sent back their samples. For the results process, we were not in a position to have this feedback. To enhance this system and avoid this limitation, we have now succeeded in the advocacy of supporting the linkage to care with a specific telephone and email service, but those developments were not available in 2016 for BaroTest.

The Health Barometer was a real opportunity to experiment with a new screening approach for HIV, HCV, and HBV based on home self-sampling among a representative and large sample of the general population of mainland France (more than 14,000 participants). It also provides a reliable assessment and update of the burden of HBV, HCV, and HIV infections in the general population in France, while reducing the costs typically associated with this type of research. The Health Barometer questionnaire also provides a wealth of information on the opinions, knowledge, and practices of the population.

With respect to the methodology used in BaroTest (ie, telephone-based survey), the acceptance rate of home self-sampling was probably different than the rate one might expect, had the invitation to participate been made through other means, for example, via the internet. Nonetheless, the data from BaroTest will contribute to better profile potential users of the home self-sampling offer, in the context of reinforcement of screening policies.

Screening assays have slightly lower performance when DBS absorbed onto filter paper than using whole blood collected through venipuncture. However, meta-analyses showed that anti-HCV and HBsAg testing using DBS compared with venous blood sampling was associated with excellent diagnostic accuracy [25]. With the same techniques and thresholds used in the BaroTest, DBS specificity and sensitivity for anti-HCV detection have recently been estimated at 98.2% and 99.1%, respectively, with the corresponding estimated values for HCV RNA detection being 100% and 98.1% [26]. For HIV, DBS sensitivity to detect HIV-seroconversion is close to that of third-generation tests (antibody detection) performed under standard conditions [27]. Therefore, the risk of not accurately detecting HIV infection—even very recent infection—during the BaroTest survey would seem limited.

We will estimate the prevalence of chronic HBV and HCV infections and HIV infection according to the participation rate and characteristics of the participants. The BaroTest will also provide information on the proportions of people infected with HCV, HBV, or HIV who are unaware of their infection that is indispensable in the context of the development of new highly effective treatments to reduce morbidity and mortality. The BaroTest results will consequently help inform new strategies for HIV, hepatitis B, and hepatitis C screening and—if the acceptability and feasibility results of the study prove conclusive—will encourage the expansion of the current screening offer to include home self-sampling.

## Abbreviations

DAAs: direct-acting antivirals
DBS: dried blood spots
EIA: enzyme immunoassay
ELISA: enzyme-linked immunosorbent assay
GP: general practitioner
HBsAg: hepatitis B virus surface antigen
HBV: hepatitis B virus
HCV: hepatitis C virus
MSM: men who have sex with men
NRC: national reference center
PBA-BSA-TW: sodium phosphate buffer containing 10% bovine serum albumin and 0.05% Tween 20
